# Histopathological characterization of hepatocellular carcinomas which are undetected by dynamic computed tomography

**DOI:** 10.1186/1476-5926-2-S1-S56

**Published:** 2004-01-14

**Authors:** Takatsugu Yamamoto, Kazuhiro Hirohashi, Katsu Sakabe, Taichi Shuto, Takahiro Uenishi, Masao Ogawa, Shogo Tanaka, Hiromu Tanaka, Shoji Kubo, Kenji Kaneda, Masami Sakurai

**Affiliations:** 1 Gastroenterological & Hepato-Biliary-Pancreatic Surgery, Osaka City University Graduate School of Medicine, Osaka, Japan; 2 Senior Health Facility Bellflower, Osaka City University Graduate School of Medicine, Osaka, Japan; 3 Pathology, Osaka City University Graduate School of Medicine, Osaka, Japan

## Introduction

A recent progress in imaging techniques like interventional radiography enables more accurate diagnosis of small hepatocellular carcinomas (HCCs). Most of HCCs are visualized by dynamic computed tomography (dynamic CT) and CT during arteriography/arterial portography (angio CT). Some early HCCs are, however, invisible in dynamic CT or angio CT [1,2]. In this study, we investigated histopathological features of HCCs not detected in dynamic CT.

## Methods

Liver specimens were obtained from 154 patients (132 men and 22 women, 29–82 years old) with small HCCs in a diameter of less than 3 cm by the surgical resection or centetic therapy. We chose 207 nodules which developed in either solitary or multicentric fashions.

Dynamic CT images were obtained with an X-Vigor (Toshiba, Tokyo) by scanning the liver in 7-mm thickness. After administration of 100-ml iopamidol, scanning was conducted at 30 sec (early phase) and 150 sec (late phase). Diagnosis was done by at least two radiologists. Tumor size was measured macroscopically. Grade of differentiation (well, moderate, poor) [3], growth pattern (expansive, replacing) [4], and the presence/absence of fibrous capsule and intratumoral Glisson's sheath were pathologically examined.

For the statistic analysis, –^2 ^or Fisher's exact test were used.

## Results

Among HCCs examined, some were not visible in both early and late phases of dynamic CT, only being found by the intra or preoperative ultrasonics or pathologic examination (= "not-detected" tumors), while others were visible either in early or late phase (= "detected" tumors). There was a significant correlation between dynamic CT images and pathological figures of fibrous capsule, intratumoral Glisson's sheath, growth pattern and grade of differentiation (Table 1). Tumors with the fibrous capsule, no Glisson's sheath and expanding growth were demonstrated to be largely poorly/moderately-differentiated HCCs, and usually detected in dynamic CT. Those with no fibrous capsule, Glisson's sheath and replacing growth, on the other hand, included both "not detected" and "detected" HCCs, and were usually well differentiated HCCs.

**Table 1:** Correlation with dynamic CT detection with pathological features

	Fibrous capsule*		Glisson sheath*	
Dynamic CT	Absent	Present	Present	Absent

Not detected	25	2	27	12
Detected	54	96	27	137

	Growth pattern*		Differentiation*	

Dynamic CT	Replacing	Expanding	Well	mod/por
Not detected	29	3	37	2
Detected	34	122	38	126

The number of nodules is indicated. * p &lt; 0.01.

Well differentiated HCCs were further divided into four types according to the growth pattern and intratumoral Glisson's sheath, and the number and mean diameter were examined (Table 2). Although the proportions of four types in each group of "not detected" and "detected" HCCs were similar, HCCs with Glisson's sheath (-)/expanding growth were more frequent in the latter compared to the former. It was also noted that the tumors with Glisson's sheath (-)/replacing growth were more frequent than those with Glisson's sheath (+)/expanding growth.

**Table 2:** The number and mean (+SD) diameter of "not detected" and "detected", well differentiated HCCs

	Glisson sheath	
	+	-
**Not detected:**		
Replacing	22 (79%); 10.1 – 3.4 mm	5 (18%); 7.8 – 5.5 mm
Expanding	0 (0%)	1 (4%)
**Detected:**		
Replacing	20 (63%); 19.0 – 6.2 mm*	6 (19%); 16.7 – 5.3 mm**
Expanding	1 (3%)	5 (16%); 24.0 – 5.5 mm

*p &lt; 0.01, **p &lt; 0.05 vs. "not detected" HCCs

## Discussion

The present study demonstrated that HCCs undetectable in dynamic CT were usually well differentiated HCCs with intratumoral Glisson's sheath, no fibrous capsule and replacing growth, while "detected" HCCs included both well and moderately/poorly-differentiated tumors. The fact that tumors were not detected by dynamic CT indicates that they were supplied with both the portal veins and hepatic arteries as in the normal liver parenchyma. Consistent with this, "not detected" HCCs usually had intratumoral Glisson's sheath and showed replacing growth, keeping a direct connection between the tumorous microvessels and sinusoids of surrounding liver parenchyma, and, furthermore, "detected" moderately/poorly-differentiated HCCs lacked these pathological features.

It was also demonstrated here that some well differentiated HCCs with intratumoral Glisson's sheath and replacing growth were detected by dynamic CT. They were larger in size than "not detected" tumors. This finding suggests that alterations to the blood supply to the tumor as demonstrated by dynamic CT images may occur during the growth of tumors, preceding the changes in the architecture of microvessels. We previously demonstrated that angio-architecture of well differentiated HCCs was similar to that of normal liver parenchyma and considered that this may be related to mature tumor cells [5] because mature tumor cells seem to retain some metabolic functions of hepatocytes and therefore require the normal angio-architecture. The mechanism by which the portal blood flow reduces during the transition from "not detected" to "detected" HCCs needs to be further clarified. It was also noted here that HCCs with Glisson's sheath (-)/replacing were higher in frequency than those with Glisson's sheath (+)/expanding growth, suggesting that disappearance of Glisson's sheath from the tumor may precede the transition from replacing growth to expanding growth.

In conclusion, "not detected" small HCCs are largely well differentiated tumors. However, as they grow larger, they become positive in dynamic CT, indicating the preferential arterial blood supply to them, in spite of replacing growth and the presence of intratumoral Glisson's sheath.
